# Intuition in Occupational Therapists’ Clinical Reasoning: A Scoping Review

**DOI:** 10.1177/15394492241300604

**Published:** 2024-12-24

**Authors:** Vermeulen P, Lavoie P, Moreau E, Rochette A

**Affiliations:** 1 University of Montreal, Canada; 2 Centre for Interdisciplinary Research in Rehabilitation of Greater Montreal (CRIR), Canada; 3 Montreal Heart Institute, Canada

**Keywords:** occupational therapy, intuition, clinical reasoning, clinical competence, knowledge

## Abstract

This scoping review aimed to map the various facets of intuition in occupational therapy (OT), from its definitions, theoretical frameworks, epistemological paradigms to practical applications, highlighting its role in decision-making. Following the Joanna Briggs Institute methodology, a systematic search of five databases from 1990 to August 2023 identified 337 records related to OT and intuition. After removing duplicates and applying eligibility criteria, 22 studies were included. Two independent reviewers conducted the title/abstract and full-text screening. Thematic analysis synthesized descriptions of intuitive reasoning, and the studies’ epistemologies were interpreted based on stated methodologies and knowledge conceptions. Key themes depicted OT’s intuition as personalized knowledge developed through practice. Constructivist paradigms recognizing subjective meaning-making predominated (63.6%), while postpositivists related to self-reported intuition to decision outcomes quantitatively (22.7%). Despite increasing interdisciplinary attention, occupational therapists’ intuition remains understudied. Integrating analytical and intuitive practice through reflection is crucial for client-centered expertise.

## Plain Language Summary

Intuition In Occupational Therapists’ Clinical Reasoning: A Scoping Review

Purpose: This scoping review aimed to map the various facets of intuition in occupational therapy (OT), from its definitions, theoretical frameworks, epistemological paradigms to practical applications, highlighting its role in decision-making. Methods: Following the Joanna Briggs Institute methodology, a systematic search of five databases from 1990 to August 2023 identified 337 records related to OT and intuition. After removing duplicates and applying eligibility criteria, 22 studies were included. Two independent reviewers conducted the title/abstract and full-text screening. Thematic analysis synthesized descriptions of intuitive reasoning, and the studies’ epistemologies were interpreted based on stated methodologies and knowledge conceptions. Results: Key themes depicted OT’s intuition as personalized knowledge developed through practice. Constructivist paradigms recognizing subjective meaning-making predominated (63.6%), while postpositivists related to self-reported intuition to decision outcomes quantitatively (22.7%). Conclusion: Despite increasing interdisciplinary attention, occupational therapists’ intuition remains understudied. Integrating analytical and intuitive practice through reflection is crucial for client-centered expertise.

## Introduction

Clinical reasoning, the cognitive process allowing occupational therapists to plan, implement and evaluate the services offered to their clients, is at the heart of occupational therapy (Shafaroodi et al., 2014). This process is based on the analysis of data and on a holistic understanding of the client in context (Mattingly & Fleming, 1994). Clinical decision-making and clinical reasoning are often used interchangeably (Higgs, 2018). However, clinical reasoning encompasses the whole thought process while clinical decision-making focuses on the final choice of action (Higgs & Jensen, 2019). Thus, clinical reasoning precedes the steps leading to decisions while decision-making consists of selecting a course of action among alternatives (Schell & Schell, 2008).

Some authors have highlighted the role of tacit, embodied, and somatic knowledge in occupational therapists’ expertise (Carrier et al., 2010; Rappolt, 2003). This practical know-how guides therapeutic actions through an intuitive understanding that goes beyond rational discourse (Greenhalgh et al., 2008). S. E. Dreyfus and Dreyfus (1980) also argue that intuition develops with expertise. Similarly, Schön (2017) describes how experts demonstrate know-how-in-action, acting intuitively without always being able to explain their reasoning.

Thus, intuition is closely linked to expertise and tacit knowledge. Nevertheless, the few studies on intuition in occupational therapy limit understanding of its potential contributions in practice. The challenge would be appreciating this experiential mode of thinking critically and reflexively, whether in education or clinical practice. To do this, it seems important to clarify its very nature within occupational therapists’ reasoning. Although there is no interdisciplinary consensus on a single definition of intuition, most conceptualizations share common aspects. First, intuition involves a fast, holistic and nonconscious mode of thinking, contrasting with slow, sequential analytical reasoning (Kump, 2022; Sinclair & Ashkanasy, 2005). Most descriptions of intuition include “rapid perception, lack of awareness of the process engaged, concomitant presence of emotions and holistic understanding of the problem situation” (Gobet & Chassy, 2008). Second, it would allow decisions to be made with little effort and in the absence of complete information by integrating multiple cues (Hogarth, 2001). Third, intuition would draw on the experience accumulated by an individual to generate relevant global impressions despite ambiguity (Nalliah, 2016). Moreover, intuition would involve the rapid formation of global associations between different elements of a situation, based on feelings and holistic impressions (Dane & Pratt, 2009). In addition, intuitive orientation has been linked to recognizing and expressing emotions in the workplace (Downey et al., 2006), suggesting that intuition can play a role in managing interpersonal relationships and effective communication within work teams.

In medicine, the use of intuition in physicians’ decisions is subject to two contrasting streams of thought (Vanstone et al., 2019), that of intuition as a potential source of errors (Chisholm et al., 2000; Croskerry, 2013) and that of its crucial role in recognizing and establishing complex diagnoses (Brink et al., 2019; Patel & Itri, 2022; Srivastava & Grube, 2009). As a result, some favor a more analytical, evidence-based approach by warning against excessive reliance on “medical instinct” (Croskerry & Norman, 2008; Kahneman et al., 1982) to avoid heuristics that can lead to biases and errors in thoughts or actions (Croskerry, 2013; Marewski & Gigerenzer, 2012). In contrast, others value clinical intuition developed through experience as a key element of clinicians’ practical professional knowledge, often referred to as “clinical intelligence” (Gauld et al., 2023; Groopman & Prichard, 2007; Higgs & Titchen, 1995; Philipp et al., 1999). This knowledge, which includes tacit and somatic aspects, is essential for rapid and competent decision-making in a constantly evolving care environment. Intuition was recognized early on as a central element of clinical decision-making in nursing. Expert nurses would develop clinical intuition that allows them to quickly grasp subtle cues and integrate various sources of information to make informed decisions (Benner, 1984; O’Neill et al., 2005; Price et al., 2017). This intuition is rooted in a deep, holistic understanding of the client, sometimes beyond explicit knowledge.

Krishnan (2018) synthesized these epistemological differences in two opposing models. The systematic-positivist model is based on analytical and cognitive reasoning, using scientific evidence and objective knowledge. It does not consider emotional and social aspects, favoring the transferability of decisions. On the other hand, the intuitive-humanistic model promotes intuition and practical wisdom, allowing the use of tacit knowledge derived from experience. Based on a subjective perspective, this model considers knowledge as resulting from individual experience. Thus, the systematic-positivist model focuses on objective analysis based on scientific evidence, while the intuitive-humanistic model emphasizes intuition and tacit knowledge derived from personal experience.

While the role of intuition in clinical reasoning is debated in the health professions, its specific application and understanding in occupational therapy remain unclear. The field has seen limited research directly addressing the role of intuition in occupational therapy. Considering the significance of clinical reasoning and decision-making in this area, this gap indicates a need for an extensive review and synthesis of the literature concerning intuition in occupational therapy. Such a synthesis would involve a systematic examination of the available research, focusing on summarizing the key concepts, methodologies, epistemological foundations, and theoretical perspectives related to intuition in this context. Undertaking this research would shed light on the current understanding of intuition within occupational therapy and how it has been approached so far. This work could significantly enhance the profession’s understanding of intuition’s role in clinical reasoning and decision-making, offering a foundation for future research and practice development in occupational therapy.

### Objective and Research Questions

This scoping review aimed to map the various facets of intuition in occupational therapy, from its definitions to its practical applications, while highlighting its role in clinical reasoning and decision-making. More specifically, this scoping review sought to answer the following questions:

1. What are the definitions of intuition in occupational therapy? What are its main characteristics and its roles in occupational therapists’ clinical reasoning?
2. What theoretical frameworks and epistemological paradigms underlie the study of intuition in occupational therapy?
3. What methodological approaches and tools have been used to study intuition in occupational therapy?

### Material and Methods

A scoping review was conducted in accordance with the Joanna Briggs Institute methodology (Peters et al., 2015; Peters et al., 2020), which is based on guidelines developed by Arksey and O’Malley (2005) and by Levac et al. (2010). Scoping reviews allow for mapping the key concepts underpinning a field of research and creating hypotheses for further research and applications in practice. Thus, a scoping review was deemed the most appropriate form of evidence synthesis, as it incorporates a broader scope and less restrictive inclusion criteria than a systematic review. A preliminary search was conducted in June 2023 in Scopus, Google Scholar, the Cochrane Database of Systematic Reviews, and the Joanna Briggs Institute (JBI) database. No ongoing or previously published scoping reviews on this topic were identified, confirming the need for this review. The search strategy was then refined and expanded to ensure comprehensive coverage of the literature.

### Inclusion Criteria

The inclusion criteria outlined below build on population, concept, and context mnemonic.

#### Population

The target population of this scoping review consisted of occupational therapists and occupational therapy students. Occupational therapy is a client-centered health profession that aims to promote health and well-being through occupation. The main goal of occupational therapy is to enable people to participate in activities of daily living. Occupational therapists achieve this by working with individuals and communities to enhance their ability to engage in the activities they want to, need to, or are expected to do, or by modifying the occupation or environment to support their occupational engagement (World Federation of Occupational Therapists, 2012) Occupational therapy assistants were excluded from this review because they work under the guidance and supervision of occupational therapists, implementing decisions made by occupational therapists and thus lacking the full independence to make decisions. Meanwhile occupational therapists and occupational therapy students autonomously conduct evaluations and make decisions regarding interventions, even if for students, these decisions may solicit guidance and supervision of a preceptor.

#### Concept

The main concept of this review was intuition in clinical reasoning and decision-making in occupational therapy. Since the concept has been little studied in occupational therapy, the search was carried out considering the interdisciplinary and multi-epistemological definitions mentioned above.

#### Context

In this scoping review, all practice settings were considered, such as hospitals, rehabilitation clinics, long-term care centers. Academic settings were also included.

#### Other

Articles written in French or English were included in the analysis. Systematic reviews were not included, but relevant citations were incorporated. This allowed capturing key intuition concepts from the broader literature, aligning with the scoping review’s comprehensive mapping approach, despite limitations evaluating study quality. There were no restrictions on the publication date. In addition, gray literature, such as unpublished theses and dissertations that met the criteria of the research question, has also been included.

### Article Search

The following databases were searched in August 2023:

- Medline on Ovid, from 1946 to present (daily update).
- Embase on Ovid, from 1980 to present (daily/weekly update).
- PsycINFO on Ovid, from 1806 to present (weekly update).
- Education Resources Information Center (ERIC).
- Occupational Therapy Systematic Evaluation of Evidence (OTseeker) (http://www.otseeker.com/).
- Physiotherapy Evidence Database (https://www.pedro.org.au/).

A combination of keywords and search terms, such as “intuition,” “intuitive,” “clinical reasoning,” and “occupational therapy” were used to identify relevant articles. An example of a Medline search strategy is shown in Table 1. In addition to the major academic databases, gray literature searches were conducted in ProQuest and Google Scholar, as these platforms are known to index a wide range of unpublished material relevant to the topic. Other gray literature repositories were not included as the research team determined the selected sources would provide sufficient coverage of the available gray literature on intuition in occupational therapy.

**Table 1.** Example of a Medline Search Strategy.

Ovid MEDLINE(R) ALL <1946 to August 24, 2023>		
1	Occupational Therapy/	15,075
2	Occupational Therapists/	746
3	occupational therap* or ergotherap*).ab, kf,ti.	19,096
4	1 or 2 or 3	24,546
5	Intuition/	1,547
6	(intuit* or hunch* or gut feeling* or tacit knowledge* or emotional intelligence).ab,kf,ti.	36,147
7	5 or 6	36,516
8	Decision-Making/	105,251
9	exp Clinical Decision-Making/	16,289
10	exp Problem Solving/	30,674
11	(decision* making or clinical thinking or clinical reason* or clinical decision* or problem* solving or diagnos* reason* or reason* strateg*).ab, kf,ti.	252,216
12	8 or 9 or 10 or 11	340,361
13	7 or 12	373,727
14	4 and 13	1,169
15	4 and 7	48

### Article Selection

Search results were imported into Covidence (Veritas Health Innovation). Duplicates were removed. Two authors (PV and EM) independently conducted the study selection in two stages, in accordance with JBI recommendations (Peters et al., 2015): (a) careful reading of titles and abstracts to assess the relevance of references according to the detailed eligibility criteria, and (b) full-text analysis of relevant articles to confirm final eligibility against inclusion criteria.At each stage, disagreements were resolved by discussion until a consensus was reached. When consensus could not be reached, a third author (AR) was consulted to arbitrate the decision. All decisions regarding inclusion and exclusion were documented in Covidence. Reasons for exclusions were noted for each reference during the article selection steps.

As this was a scoping review, no formal critical appraisal of the included studies was conducted, as this is not a common practice within this type of evidence synthesis methodology (Peters et al., 2020).

### Data Extraction and Mapping

To describe the articles included in this scoping review, data extraction tables were used to collect information on study characteristics: country, research design, participants, methods, and tools used to study intuition, study purpose and epistemological approach.

A thematic analysis was performed following the coding steps recommended by Paillé and Mucchielli (2021) to answer the first research question of defining the concept of intuition. The coding grid was created based on two articles and completed iteratively by reading all the articles. The subthemes were identified before grouping them into themes. A descriptive analysis completed the data extraction for the definitions of intuition. Table 3.1 in supplemental appendices shows descriptive data extracted for the nature, definition, tools, and evaluation of intuition.

To answer the second research question, an inductive three-step approach was carried out to identify the theoretical frameworks and paradigms used to define intuition, based on the work of Omodan and Addam (2022), Crotty (1998) and Creswell and Creswell & Creswell (2017). First, data on the methods were extracted, and research approaches were categorized (qualitative, quantitative, mixed, or theoretical articles). Second, the objectives and theoretical frameworks of the studies were analyzed to identify conceptions of knowledge and research goals. Third, these elements were connected to the epistemological paradigms Crotty (1998) described, such as positivism, postpositivism, and constructivism. This interpretive approach allowed positioning the studies in relation to the major epistemological currents that influence knowledge production. Potential biases linked to this interpretation of the implicit philosophical foundations were mitigated by discussions between team members.

Finally, to answer the third question, a mapping of methodological approaches and tools used to study intuition was performed using a data extraction form developed to compile methods of evaluating intuition and tools used.

## Results

### Study Selection

**Figure 1.**
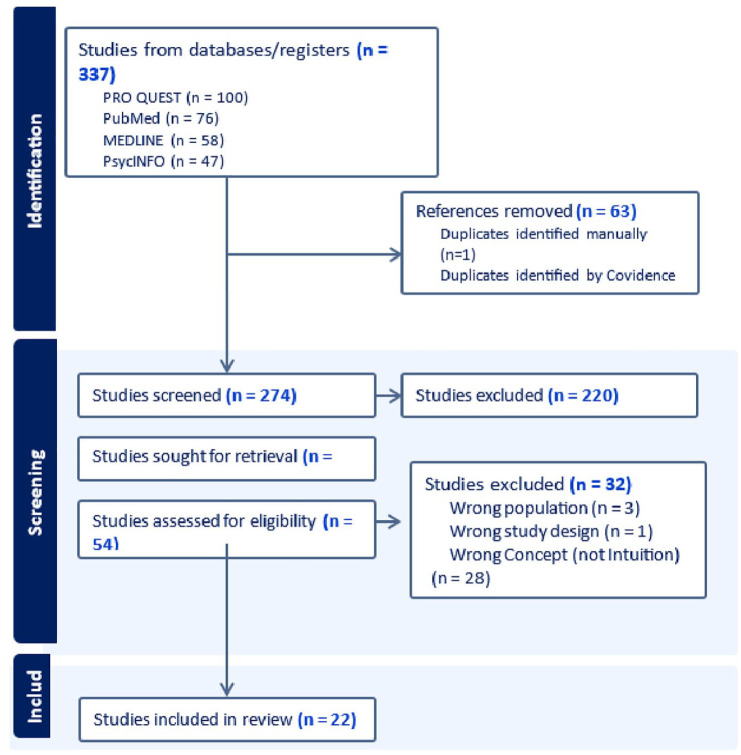
Study Selection Flow Diagram.

Figure 1 presents the flow diagram of the selection process. The initial search generated 337 records. After removing 63 duplicates, 54 articles (19.7%) were selected for full-text analysis. The authors then excluded 32 articles (59.3%) during the full-text review, notably due to the lack of focus on the concept of intuition. To ensure reliability of the article selection process, the kappa coefficient was calculated and found to be 0.69, indicating a substantial level of agreement between the reviewers.

### Description of Articles

Table 2 presents the characteristics of the included articles. Six articles were published between 1990 and 2000, 12 between 2001 and 2011, and four between 2012 and 2023. The articles are distributed across five different countries, with the majority coming from the United States (*n* = 7/22) and Australia (*n* = 6/22). Over a third of the articles are theoretical (*n* = 8/22), and four are from the gray literature (including two book chapters, one doctoral thesis, and one master’s thesis). Although occupational therapy students were included in the search for studies, only articles concerning occupational therapists met the selection criteria.

**Table 2.** Characteristics of Selected Articles (n = 22) Presented by Year of Publication.

Reference	Design	Methods and sample size (OT)	Aims	Paradigm
Grime (1990) United Kingdom	Theoretical article	N/A	To examine the decision-making process involved when receiving a referral as a community occupational therapist.	Constructivism
Mattingly and Fleming (1994) USA	Theoretical article	N/A	To propose an alternative to the medical model of clinical reasoning, which is an interpretive, or meaning-centered, model that focuses on how patients make sense of their disability and its meaning for their individual lives.	Constructivism
Hooper (1997) USA	Qualitative single-case study	Semi-structured interviews and an observation of a filmed treatment session (*n* = 1)	To explore the relationship between pretheoretical assumptions and clinical reasoning in occupational therapy practice.	Constructivism
Roberts (1996) United Kingdom	Theoretical article	N/A	To explore the nature of reasoning about clinical problems in occupational therapy and to emphasize the universality of the basic process of reasoning.	Cognitivism
Colditz (2000) USA	Theoretical article	N/A	To explores the importance of passion and intuition in the field of hand therapy.	Pragmatism
Harries and Harries (2001) USA	Theoretical article	Focus Ethnographic and information-processing approaches with TIntS, the Big Five Aspect Scales (BFAS), and the Miller Intuitiveness Instrument	To critique the reasoning studies of the 1980s and 1990s, focusing on ethnographic and process-tracing approaches, and to identify a need for an approach that acknowledges the experienced thinker’s intuitive reasoning.	Constructivism
Leicht and Dickerson (2002) USA	Theoretical article	N/A	To provide an overview of the concept of clinical reasoning in occupational therapy.	Pragmatism
Mekkes (2003) USA	Qualitative phenomenology (Master Thesis)	In-depth interviews and thematic analysis (*n* = 3)	To understand the influence of occupational therapists’ worldview on their professional practice and exploring the link between their worldview and clinical reasoning.	Constructivism
Rassafiani et al. (2006) Australia	Quantitative cross-sectional study	Quantitative data with Social Judgment Theory (*n* = 12)	To identify clinical expertise in occupational therapy based on decision-making characteristics.	Postpositivism
Rassafiani et al. (2008) Australia	Quantitative cross-sectional descriptive study	Case vignettes (*n* = 18)	To identify the factors influencing the decision-making process of occupational therapists in managing upper limb hypertonia in patients with cerebral palsy.	Postpositivism
Rassafiani et al. (2009) Australia	Quantitative quasi-experimental study	Case vignettes (*n* = 18)	To identify the decision-making characteristics of expert occupational therapists.	Postpositivism
Williams and Paterson (2009) Canada	Qualitative phenomenology	Semi-structured interview and participant reflection (*n* = 3)	To explore the concept of professional artistry in occupational therapy.	Constructivism
Vachon (2009) Canada	Qualitative case study (PhD Thesis)	Individual interviews, focus group and thematic analysis (*n* = 8)	To explore the use of a reflective approach to integrate evidence-based practice into occupational therapy practice in work rehabilitation.	Constructivism
Chaffey et al. (2010) United kingdom	Qualitative grounded theory	Semi-structured interview (*n* = 9)	To study how mental health occupational therapists rely on intuition in their clinical practice, emphasizing the importance of patient interaction and connection in their decision-making process.	Constructivism
Metzler and Metz (2010) Canada	Theoretical article	Analysis with Knowledge-To-Action Process	To examine the concept of knowledge translation in occupational therapy and highlight the value of client, therapist, and research knowledge.	Constructivism
Vachon et al. (2010) Canada	Qualitative Case Study	Individual interviews, focus group and thematic analysis (*n* = 8)	To investigate the clinical decision-making methods, they employed and elucidate the empowerment process they established in order to transition into evidence-based practitioners.	Constructivism
Unsworth (2011) Australia	Gray literature	N/A	To examine an occupational therapist’s use of clinical reasoning when accompanying stroke patients with cognitive and perceptual issues.	Constructivism
Chaffey et al. (2012) Australia	Quantitative cross-sectional survey	Self-administered questionnaire (*n* = 134)	To investigate the relationship between intuition and emotional intelligence among occupational therapists in mental health practice.	Postpositivism
Pretz et al. (2014) USA	Quantitative cross-sectional survey	Types of Intuition Scale (*n* = 23)	To introduce the creation and validation of a novel assessment tool for intuition, known as the Types of Intuition Scale (TIntS), designed to evaluate three specific forms of intuition: holistic, inferential, and affective.	Postpositivism
Higgs (2019) Australia	Gray literature	N/A	To explore the concept of practice wisdom in professional practice.	Constructivism
Fried (2020) Israël	Theoretical article	N/A	To emphasize the importance of both acquired knowledge and clinical experience in the treatment of patients, and to highlight the role of courage in recognizing one’s own limitations as a therapist.	Constructivism
Pickens et al. (2022) USA	Qualitative study	Focus groups with HESTIA app using the uMARS usability (*n* = 8 OT and 1 PT)	To identify challenges faced by home evaluators when conducting safety assessments, to explore the perspectives of experienced and early career practitioners on the content and usability of home safety assessments, and to identify best practices for home safety evaluations.	Constructivism

*Note.* OT = Occupational Therapist, PT = Physiotherapist, USA = United States of America.

### Definitions and Roles of Intuition

Intuition in occupational therapists’ clinical reasoning would be an unconscious, nonsequential information processing mode resulting in “direct knowing without any use of conscious reasoning” (Higgs, 2019). This conceptualization captures the tacit, experiential qualities of intuition, which involves a blend of tacit knowledge, experience, emotions, and sensations to enable accurate judgments despite incomplete information (Chaffey et al., 2010; Harries & Harries, 2001). However, this definition risks oversimplifying the complex interplay of conscious and unconscious processes underlying intuitive thinking. A critical analysis of the varied definitions of intuition in occupational therapy could help elucidate its multidimensional nature. Intuition may in fact integrate sensations, emotions, and past experiences in ways that are not readily articulable. A more nuanced understanding, examining distinctions between types of intuition such as holistic, inferential, and affective (Pretz et al., 2014), could provide richer insights. Exploring how these different facets of intuition manifest in occupational therapists’ clinical reasoning and decision-making could provide valuable perspectives.

The thematic analysis resulted in four themes: intuition as (Theme 1) personalized knowledge, (Theme 2) a valuable decision-making tool, (Theme 3) a source of creativity and joy, and (Theme 4) the limits of intuition.

1. *As personalized knowledge, intuition would rely on an occupational therapist’s accumulated clinical experience and ability to draw implicit connections (Theme 1).*

Intuition in clinical reasoning would rely on the occupational therapist’s accumulated experience and tacit knowledge developed over years of practice (Harries & Harries, 2001). It demonstrates itself through instant judgments made without conscious, sequential reasoning (Higgs (2019). The knowledge behind intuitive judgments has often been ingrained to the point of being difficult to articulate explicitly (Chaffey et al., 2010).

An occupational therapist’s personalized knowledge shapes the accuracy and relevance of their intuitions. Greater expertise comes with more reliable, intuitive decision-making that swiftly draws connections between a presenting situation and past cases (Rassafiani et al., 2009). Intuition supplements clinical data by filling gaps in understanding the client’s unique perspectives and contexts (Fried, 2020; Leicht & Dickerson, 2002). It combines professional knowledge with personal qualities like emotional intelligence and experience gained in the field (Pickens et al., 2022).

Overall, an individual clinician’s life journey would inform their intuitive knowing just as much as their formal education and training (Pickens et al., 2022). Long-cultivated intuition would be a crucial asset in client-centered, contextualized reasoning (Fried, 2020; Pickens et al., 2022).

1. *Intuition is a valuable decision-making tool that complements analytical reasoning by rapidly identifying patterns and enabling decisions despite uncertainty and incomplete information (Theme 2).*

While not infallible, intuition would serve as a valuable complement to analytical thinking, enabling occupational therapists to make rapid yet effective decisions (Harries & Harries, 2001; Rassafiani et al., 2009). Intuitive judgments would also help interpret the relevance of clinical data when time is limited (Grime, 1990).

In addition, intuition would facilitate decision-making even when details are ambiguous or incomplete (Harries & Harries, 2001; Higgs, 2019). It handles uncertainty better than strict logic by flexibly bridging gaps in understanding. However, intuition should not serve as the sole basis for clinical choices without attempting to gather more objective information. Instead, it interacts dynamically with objective data, theories, and experiential knowledge to generate optimal interventions tailored to the client’s needs (Metzler & Metz, 2010).

1. *Intuition infuses clinical reasoning with creativity, artistry, sudden insights, and joy through its nonrational and emotion-guided way of discovering solutions (Theme 3).*

Beyond enabling decisions, intuition would introduce creativity, artistry and joy to the therapeutic process (Colditz, 2000; Williams & Paterson, 2009). It would bring “constant discovery of the knowledge and skills within you” as a clinician, producing sudden insights that feel like “episodes of suddenly knowing something that you did not realize you knew” (Colditz, 2000). Intuition also utilizes emotions as part of holistic problem-solving, with feelings “implicated in the use of intuition” for creativity and decision-making (Chaffey et al., 2012). Overall, intuition would promote the professional artistry involved in individualizing client care (Williams & Paterson, 2009).

Letting intuition flourish would make OT come alive with meaning. It serves as “a bridge when sensory and intellectual processes fail to inform adequately, enhancing creativity” (Fried, 2020). The occupational therapist can then meet each client with an open, genuine spirit of exploration.

1. *While valuable, intuition has limits and risks biased judgments if solely relied upon (Theme 4).*

The reliance on intuitive reasoning alone risks biased, erroneous judgments (Higgs, 2019). While intuition evaluates information relevance, sole dependence on its unconscious knowing is ill-advised when objectivity is needed.

In addition, certain practice contexts may constrain intuitive creativity, like legally complex, risk-averse environments (Colditz, 2000). Training programs should teach therapists to balance intuitive with analytical thinking by taking time to consciously reflect before acting (Vachon, 2009). Understanding intuition’s limitations would allow capitalizing on its strengths.

### Theoretical Frameworks and Epistemological Paradigms

One study (*n* = 1/22) explicitly described its epistemological paradigm (Vachon, 2009). Thus, the findings in Table 3 reflect our analysis of the researcher positions, views of intuition, methodologies, and key results regarding intuition, as well as limits regarding the epistemological paradigms in the study of intuition.

**Table 3.** Epistemological Paradigms in the Study of Occupational Therapists’ Intuition.

Position of researcher	View of intuition	Methodology	Key results regarding intuition	Limits
Constructivism (*n* = 14)				
Subjective perspective seeking to understand individuals’ meaning making processes and perspectives. Recognizes that interpretation is shaped by personal experiences and beliefs.	Personal and contextual way of understanding the world that develops from a therapist’s experiences. It is a type of “meaning making” based on embodied knowledge and unconscious schemas built over time in practice. It interacts with the therapist’s “worldview” and assumptions that act as an interpretive filter to guide reasoning.	Qualitative methods like interviews, focus groups and ethnographic observation to capture therapists’ perspectives and experiences related to intuition. Conceptual, theoretical analysis examining intuition and clinical reasoning.	Intuition was found to be influenced by therapists’ underlying beliefs, life experiences, and assumptions about the world. It developed from accumulated practical experience working with clients over an extended time. Results highlighted the weight of interpretation and subjectivity in the intuitive reasoning process of occupational therapists.	Findings related to the subjective experience of intuition may not generalize across different settings and contexts.
Postpositivism (*n* = 5)				
Objective, independent stance not influenced by values or biases	Quantifiable and measurable psychological phenomenon that can be reliably correlated with other variables, such as clinical expertise or decision-making success.	Surveys using validated psychometric scales and statistical analysis of measurable indicators like agreement rates	Correlations between level of intuition and emotional intelligence. The client’s severity of symptoms is the most influential factor on treatment decisions.	Findings may be limited in generalizability due to student samples and recruitment issues
Pragmatism (*n* = 2)				
Values knowledge based on direct clinical experience with patients, at the same level as theoretical and empirical knowledge	Result of prolonged practical learning from patient experiences leading to expertise. Allows experienced clinicians to make rapid, effective decisions drawing on embodied knowledge.	Patient cases to demonstrate how intuition helped guide sound treatment decisions. Personal conceptual reflections	Intuition allowed to deviate from protocols when necessary.	Risk of missing the nuanced interplay of conscious and unconscious factors underlying highly intuitive clinical judgment.
Cognitivism (*n* = 1)				
Identifies regularities and mental processes underlying clinical reasoning.	Spontaneous mental process enabling rapid decision-making.	Conceptual analyses, theoretical discussions.	Conceptualized reasoning as a problem-solving process.	Does not adequately capture the complexity and subtlety of real-world clinical decision-making.

Constructivism was the paradigm found in 63.6% of studies (*n* = 14/22). Within this paradigm, intuition is seen as a subjective, contextual way of understanding the world stemming from personal experiences. Qualitative methods explore how therapists’ underlying beliefs and assumptions shape intuitive judgments. This body of research finds intuition develops from accumulated practical knowledge, interacting with worldviews to guide reasoning.

The pragmatist paradigm (*n* = 2/22) values intuition as allowing experts to make rapid and effective decisions by drawing on embodied experiential knowledge. In contrast, the postpositivist paradigm (*n* = 5/22) seeks to measure intuition abilities and correlates empirically and objectively. Here, statistical analyses identify factors influencing clinical decisions, with client difficulty being most impactful. However, the subjective, contextual nature of intuition may obstruct generalizations and transferability across settings. Finally, fundamental disparities emerge around whether intuition is studied as a personal meaning-making process versus an objectively quantifiable skill. Studies grounded in constructivism, such as Mekkes (2003), conceptualize intuition as personal meaning-making arising from the occupational therapist’s contextual experience. In contrast, postpositivist studies like Pretz et al. (2014) tend to quantify intuition as a measurable psychological construct for generalization.

However, 13 studies (59.1%) cited theoretical concepts to guide their work and understanding of intuition (Table 4). A total of eight theoretical concepts were cited in the studies, most of which are coming from cognitive psychology (*n* = 6/8).

**Table 4.** Summary of Theoretical Concepts Used in the Study of Intuition.

Theoretical concept	References	Definition	Discipline
Dreyfus and Dreyfus’ stage theories of cognitive development	Mekkes (2003), Roberts (1996), (Rassafiani et al., 2006), Mattingly and Fleming (1994), Vachon (2009) *	Thinking progresses from more analytical to more intuitive with experience.	Cognitive psychology
Schema theory	Roberts (1996)	Intuition in reasoning is related to the retrieval of stored knowledge and how knowledge is represented and used.	Cognitive psychology
Cognitive continuum theory/ Framework	Grime (1990), Roberts (1996), (Leicht and Dickerson, 2002), Pretz et al. (2014) *	Hammond’s 1988 cognitive continuum contrasts technical-rational analysis with intuition and asserts that intuition is a legitimate mode of inquiry in decision-making.	Cognitive psychology
Pattern recognition	Roberts (1996) *, Chaffey et al., (2010),	Role of pattern recognition in intuition, where practitioners intuitively retrieve and apply relevant schemata to generate hypotheses and make decisions.	Cognitive psychology
Cognitive processes	Pretz et al. (2014), (Rassafiani et al., 2008), (Chaffey et al., 2010), (Chaffey et al., 2012)	Intuitive reasoning can be contrasted with analytical thinking, suggesting that both are essential in clinical reasoning.	Cognitive psychology
Ethnographic and information-processing methods	(Harries and Harries, 2001)	Limitations of the information-processing methods in accessing unconscious and rapid reasoning at the intuitive end of the continuum.	Cognitive psychology
Reflective practice	Vachon (2009), Vachon et al. (2010), Chaffey et al. (2012) *	Reflection is a key element in the clinical reasoning of expert therapists, contributing to self-awareness and the development of the therapeutic alliance.	Education and psychology
Intuitive ethics	Higgs (2019)	Concept of intuitive ethics as an ethical theoretical perspective that supports practice wisdom.	Ethical philosophy

* The approach was named without further referring to it later in the analysis.

### Methodological Approaches and Tools Used

Of the 22 studies, 13 studies used a variety of methodological approaches to study intuition empirically: seven qualitative studies are based primarily on semi-structured interviews and thematic analysis (Chaffey et al., 2010; Hooper, 1997; Mekkes, 2003; Unsworth, 2011; Vachon, 2009; Vachon et al., 2010; Williams & Paterson, 2009), three cross-sectional quantitative studies used self-report questionnaires (Chaffey et al., 2012; Harries & Harries, 2001; Pretz et al., 2014), and three quantitative studies were based on occupational therapists’ analysis of clinical vignettes (Rassafiani et al., 2006; Rassafiani et al., 2008, 2009).

Two articles used validated tools to study intuition (*n* = 2/22). Chaffey et al. (2012) et Pretz et al. (2014) both used the Cognitive Style Index (CSI) to measure cognitive style and the Self-Report Emotional Intelligence Test (SUEIT) to measure emotional intelligence. The authors studied the relationship between these two constructs using statistical analysis (Spearman’s correlation) based on scores obtained via these self-administered instruments in a sample of 208 occupational therapists. Only one study characterized types of intuition (Pretz et al., 2014), reusing the Types of Intuition Scale that defines holistic, inferential, and affective intuition. Precisely, “holistic intuitions are judgments based on a qualitatively non-analytical process” while “inferential intuitions are judgments based on automated inferences” that were once analytical, and “affective intuitions are judgments based primarily on emotional reactions” (Pretz et al., 2014). This scale has been administered in several quantitative studies aimed at establishing its internal consistency, temporal stability and construct validity, by examining its correlations with other measures of intuition and personality.

In both articles, intuition was studied from a postpositivist perspective through quantitative methods, with correlational or quasi-experimental designs using standardized self-administered instruments to quantitatively measure intuitive abilities and statistically relate them to other constructs.

Finally, eight of 22 articles did not mention a specific methodological approach nor used any tools as they were theoretical articles (Colditz, 2000; Fried, 2020; Grime, 1990; Higgs, 2019; Leicht & Dickerson, 2002; Mattingly & Fleming, 1994; Metzler & Metz, 2010; Roberts, 1996).

Furthermore, accessing intuition in evaluation requires apt tools and conditions. Gobet and Chassy’s Template Theory offers promise by modeling how seasoned practitioners intuitively draw from accumulated “templates” of meaningful patterns (Pickens et al., 2022) *(Method)*. “Therapists needed to be aware of and understand their emotions to access intuition, to trust their emotions to act on them, and to use their emotions in problem solving and decision making” (Chaffey et al., 2012). Explicit reasoning has boundaries in articulating artful expertise central to contextualizing client needs (Harries & Harries, 2001) *(Factors influencing measurement)*.

## Discussion

The aim of this scoping review was to define the concept of intuition, identify the theoretical underpinnings that support it, and map the methodological approaches and tools to study it. The results show that, despite the growing interest in intuition in other medical professions, this concept remains little explored in occupational therapy with only 22 articles found on this topic since 1990.

### Definition of Intuition in Occupational Therapy

The results of this scoping review highlight a consistent definition of intuition across occupational therapy studies. Overall, intuition represents rapid reasoning rooted in experience-based knowledge and emotional perceptions at the unconscious level. This conceptualization corroborates definitions found in other disciplines, notably nursing and psychology. For example, P. Hassani, A. Abdi, R. Jalali, & N. Salari’s (2016) phenomenological study of 12 nurses describes intuition as “ immediate unconscious perception [and] direct understanding of truths. One of the aspects that differentiates the definition of intuition in occupational therapy from other professions is its explicit relationship with expertise and tacit clinical knowledge developed through experience. As Schön (2017) and Higgs and Titchen (1995) explained, expert practitioners would acquire embodied practical knowledge that is difficult to verbalize, but which guides their actions intuitively. At the conceptual level, only one study distinguished between different types of intuition—holistic, inferential and affective (Pretz et al., 2014). An interesting perspective would be to explore this distinction further. This would allow a better understanding of the processes underlying expert intuition in occupational therapy.

Delving into intuition definitions across disciplines reveals alignment but also opportunities for occupational therapy. Medicine describes clinical intuition as drawing on physicians’ accumulated experiential knowledge to understand patients’ needs (Benner et al., 2008). This aligns with occupational therapy depictions. However, nursing offers multilayered ethical models of intuition, like “knowing the patient” and “moral imagination” (P. Hassani, A. Abdi, & R. Jalali, 2016; Rew, 2000). And psychology extensively categorizes intuition types, contrasting with minimal type differentiation in occupational therapy (Gore & Sadler-Smith, 2011; Tonetto & Tamminen, 2015). Overall, while occupational therapy shares the view of intuition as expertise-based reasoning, exploring ethical, relational, and nuanced aspects found in nursing and psychology models could enrich understanding. As Cappelletti et al. (2014) describe, comparing reasoning across professions reveals valuable nuances. Occupational therapy has potential to expand conceptualizations by learning from other fields.

### Link Between Intuition Definitions and Epistemology

Results showed intuition definitions are partially shaped by the researchers’ epistemological paradigms. Studies grounded in constructivism conceptualize intuition as personal meaning-making arising from the occupational therapist’s contextual experience. By contrast, postpositivists tend to quantify intuition as a measurable psychological construct for generalization (Hooper, 1997; Pretz et al., 2014). This study also highlights the marked influence of constructivist paradigms, which consider reality and knowledge as social constructions shaped by the meaning actors give to their experiences (Thomas et al., 2014). By adopting an interpretive stance, studies thus sought to capture occupational therapists’ subjective perspectives on the intuitive process and its role in their reasoning. This primacy given to understanding the phenomenon of the participants’ point of view is consistent with the phenomenological roots of occupational therapy as a client-centered profession (Finlay, 2001; Mroz et al., 2015) According to Crotty (1998), there is a solid link between epistemology and the definition of concepts in research. Indeed, researchers’ epistemological conceptions would influence their way of defining what constitutes plausible knowledge as well as their viewpoints on the nature of reality. This relationship was reflected in the conceptualization of intuition presented in the articles reviewed. The definition given to intuition by the authors stems from their broader epistemological paradigm regarding what constitutes valid and reliable knowledge in their field of research. Thus, the way researchers defined intuition was part of their epistemological and ontological visions of what constitutes the production of valid and legitimate scientific knowledge in their field. The authors’ ontological and epistemological conceptions therefore would influence their theoretical perspective on complex phenomena such as intuition. Overall, there was an absence of consensus around defining intuition itself in the research. Competing constructivist and postpositivist perspectives generated different representations. A dialectical approach integrating these methods could potentially yield greater insight into clinical reasoning processes.

### Link Between Epistemology and Methodology

There was a close link between the identified epistemological paradigms and the methodological choices made by researchers to study intuition. Indeed, studies based on constructivism predominantly favor qualitative methods such as semi-structured interviews, observation, and thematic analysis. These comprehensive methods would make it possible to understand the participants’ subjective perspectives and the meaning they give to their experience of intuition. In contrast, postpositivist studies used quantitative methods (surveys, statistics) to determine intuition and establish causal relationships with other variables. The influence of ontological and epistemological conceptions on methodological choices is recognized here.

Some authors in nursing science have also discussed this link between epistemology and research methods. For example, Weaver and Mitcham (2008) in their conceptual analysis of nursing, concluded that the research method used stems directly from the researcher’s foundational philosophical assumptions. Thus, the epistemological distinctions observed in the study of intuition in occupational therapy are reflected in the diversity of research designs chosen.

However, this scoping review also highlighted the lack of validated and standardized tools to specifically assess clinical intuition in occupational therapy. Indeed, most tools were developed for study purposes. This finding calls for the development and validation of intuition measurement scales adapted to the context of occupational therapy, which would combine for example mixed methods with self-reported data and behavioral or neurophysiological measures of intuitive cognition (Glöckner & Witteman, 2010).

The marked diversity of epistemological paradigms reflected in the literature on intuition in occupational therapy aligns with a constructivist perspective that values multiple, context-dependent ways of knowing. Rather than seeking consensus, this pluralism should be embraced as it enables a richer, more nuanced understanding of the complex phenomenon of intuition in clinical reasoning.

### Comparison With Other Professions

The relative lack of research on intuition in occupational therapy, compared with other health care fields, is worth noting. Disciplines such as nursing and medicine have dedicated significant attention to understanding the role of intuition in clinical reasoning and decision-making (Hassani, Abdi, Jalali & Salari, 2016; Vanstone et al., 2019). This may be due to differences in the epistemological foundations and professional cultures of these fields. For example, the client-centered, holistic approach of occupational therapy may align more closely with constructivist views of intuition as a subjective, contextual way of understanding the world. In contrast, the more positivist traditions in medicine and nursing may have led to a greater focus on measuring and quantifying intuitive abilities. Exploring these disciplinary differences in how intuition is conceptualized and studied could provide valuable insights for the occupational therapy profession.

### Implications for Occupational Therapy

This scoping review highlighted clinical intuition’s role in occupational therapists’ real-world decision-making, enabling rapid assessment, potential solutions and informed judgments despite ambiguous information (Harries & Harries, 2001; Hooper, 1997). By drawing on clinical expertise and tacit knowledge, intuitive reasoning would also facilitate personalized, creative, and practically wise interventions (Schön, h). However, some articles warn that overreliance on intuition risks bias, especially in new situations beyond the therapist’s experiential patterns (Croskerry, 2013; Grime, 1990; Higgs, 2019). Thus, developing reflexivity to recognize and articulate intuitive reasoning would be essential to critically integrate it in practice (Unsworth, 2011; Vachon, 2009; Vachon et al., 2010). Reflective strategies like mentorship and collective analysis of emerging intuitions could be implemented. Rather than view intuition as an error source as in medicine (Croskerry & Norman, 2008), its potential contribution along with analytical approaches should be promoted (Cader et al., 2005).

Despite growing attention in related fields, clinical intuition remains understudied in occupational therapy research. Exploring this phenomenon could enrich expertise development models like S. E. Dreyfus and Dreyfus (1980) staged skill acquisition theory. Methodologically, tailored tools combining self-reports, behavioral observations and neuroimaging may contribute to capture this complex construct (Glöckner & Witteman, 2010), along with longitudinal studies tracking intuitive thinking development.

Although intuition largely develops through clinical experience (Hogarth, 2001), fostering novice therapists’ abilities to recognize and articulate intuitive reasoning early on is also essential (Harries & Harries, 2001). Providing reflective opportunities to vocalize intuitive thoughts would enable self-awareness and informed judgment. In addition, incorporating authentic practice scenarios eliciting intuitive responses, while guiding complementary analytical reasoning, would prime early intuitive skills development essential in ambiguous situations (Schön, 2017). Ultimately a balanced, critically reflective approach combining analytical and intuitive practice would be key for expertise (H. L. Dreyfus & Dreyfus, 2005; Eraut, 2000).

As expertise develops, professionals learn to use both analysis and intuition in a balanced manner (H. L. Dreyfus & Dreyfus, 2005). While novices rely more on analytical rules, experts develop intuitive recognition of meaningful patterns through experience, which allows faster and more context-specific responses (Eraut, 2000; Hogarth, 2001). However, effective experts also critically reflect on their intuitions rather than taking them as facts, enabling balanced judgment (Crow et al., 1995; Rew, 2000). Fostering this reflective capability early allows developing professionals to build awareness and effectively integrate intuitive and analytical reasoning (Harries & Harries, 2001; Schön, 2017).

### Strengths and Limitations

This scoping review presents some methodological strengths conducted in accordance with the JBI methodology. The exhaustive search strategy across five databases and the rigorous selection of articles by two independent reviewers ensure the integration of relevant scientific writings on the subject. In addition, the iterative process of consultation with the team throughout the process contributes to strengthening the credibility of the results. However, some limitations should be considered in interpreting the conclusions of this analysis. First, most studies identified originate from the United States, Canada, the United Kingdom, and Australia. It would be relevant to explore whether different sociocultural contexts bring other perspectives on the nature and role of clinical intuition in occupational therapy. Second, the limited number of articles on the subject (*n* = 22) limits the depth of understanding of certain aspects such as the use of tools or the underlying mechanisms of this mode of reasoning. Conducting further research on a larger scale or by considering also occupational therapy assistants who were excluded from this review would enrich the understanding of this complex phenomenon.

## Conclusion

This scoping review highlighted the underappreciated role of clinical intuition in effective occupational therapy reasoning and interventions. Findings reveal how intuition introduces creativity, artistry, and meaning to the therapeutic process. Actively cultivating skills to identify, appraise and integrate rapid insights could strengthen novices’ reasoning. Exploring transitions were embodied knowledge guides expert practice warrant attention. An integrative pluralist approach combining constructivism and postpositivism through mixed methods could illuminate facets of intuitive reasoning. Longitudinal studies tracking students to experts may reveal analytical-to-intuitive skill development. Proactively adopting intuition skills while embracing epistemological diversity can enrich client-centered practice and knowledge advancements.

## Supplemental Material

sj-docx-1-otj-10.1177_15394492241300604 – Supplemental material for Intuition in Occupational Therapists’ Clinical Reasoning: A Scoping Review Supplemental material, sj-docx-1-otj-10.1177_15394492241300604 for Intuition in Occupational Therapists’ Clinical Reasoning: A Scoping Review by Vermeulen P, Lavoie P, Moreau E and Rochette A in OTJR: Occupational Therapy Journal of Research
