# Supplementary material for: Intuition in Occupational Therapists’ Clinical Reasoning: A Scoping Review
Source: OTJR (Thorofare N J). 2024 Dec 24;45(4):640–54. doi: 10.1177/15394492241300604 (PMC12398635; doi:10.1177/15394492241300604)
Supplement: sj-docx-1-otj-10.1177_15394492241300604 – Supplemental material for Intuition in Occupational Therapists’ Clinical Reasoning: A Scoping Review [file sj-docx-1-otj-10.1177_15394492241300604.docx]

**Supplemental Files**

**table 3.1 Extraction of data concerning the definition and evaluation of intuition**

| Concept | Sub-concept | Exemples | Article |
| --- | --- | --- | --- |
| NATURE AND DEFINITION OF INTUITION | *Diverse definitions of intuition* | ""Intuition” refers to concepts ranging from gut feelings to snap judgments to premonitions about the future." | (Pretz et al., 2014) |
|  |  | "Intuition can be defined as a non-sequential information processing mode, which comprises both cognitive and affective elements and results in direct knowing without any use of conscious reasoning." | (Higgs, 2019) |
|  |  | "The participants in this study understood intuition to be knowledge without conscious reasoning." | (Chaffey et al., 2010) |
|  |  | "Intuition is described as direct knowing without the use of conscious reasoning." | (Higgs, 2019) |
|  |  | "Intuition is elusive and ‘underground’, provides a definition of intuition and describes a hidden intuitive practice." | (Chaffey et al., 2010) |
|  |  | "The definition of intuition contained in Fig. 2, as knowledge without conscious awareness of reasoning, is consistent with other health science literature that has reported intuition to centre on knowledge that is difficult to articulate."" | (Chaffey et al., 2010) |
|  |  | "this mode was not necessarily associated with increased utilization of research evidence, and two different autonomous modes were distinguished: the intuitive and the thoughtful autonomous mode. When participants used the intuitive autonomous mode, they identified different possible solutions for the difficult practice situation and took action before assessing the appropriateness or relevance of their actions to the context. This mode allowed participants to leam and change their practice, but not according to an evidence- based practice model, since they used tacit appraisal or intuition rather than research evidence to support their clinical decision-making process." | (Vachon, 2009) |
|  |  | "Westcott (1968) described intuitive processing involving 'intuitive leaps,' the result of previously analytical processing that had become automatic with practice. That is, intuition was defined as accurate judgment in the absence of complete information. This type of intuition is inferential and characterizes expert judgment." | (Pretz et al., 2014) |
|  |  | "Intuition was found to be embedded within clinical reasoning. From the data, intuition was defined as knowledge without conscious awareness of reasoning. " | (Chaffey et al., 2010) |
|  |  | "According to Abernathy and Hamm (1995), surgical intuition is defined as 'an immediate and global understanding of the clinical situation that guides decision making.' Intuition is considered a form of clinical reasoning that is based on experience and tacit knowledge." | (Harries & Harries, 2001) |
|  |  | "Many researchers have emphasized the holistic nature of intuition. Jung (1971) described intuition as an unconscious, primary mode of perception. Intuitive individuals are inward-focused, processing information in a holistic manner, in contrast to sensate individuals whose processing is grounded in outward sensory experience." | (Pretz et al., 2014) |
|  |  | "Intuition as described by Bergson [10] is a direct consciousness, a perspective that is almost indistinguishable from the thing itself. Intuition provides us with real information that cannot be justified or proven in a conventional manner by empirical confirmation – nor is it necessary to do so. Intuition serves as a bridge in situations in which sensory and intellectual processes fail to inform us adequately." | (Fried, 2020) |
|  | *Types of intuition* | "The Types of Intuition Scale (TIntS) identifies three distinct types of intuition: holistic, inferential, and affective." | (Pretz et al., 2014) |
|  |  | "The theoretical view of intuition as three distinct types: holistic, inferential, and affective. Holistic intuitions are judgments based on a qualitatively non-analytical process, decisions made by integrating multiple, diverse cues into a whole that may or may not be explicit in nature. Inferential intuitions are judgments based on automated inferences, decision-making processes that were once analytical but have become intuitive with practice. Affective intuitions are judgments based primarily on emotional reactions to decision situations." | (Pretz et al., 2014) |
|  |  | "When the occupational therapists tended to use primarily the cautious and autonomous intuitive modes to justify their actions, the reflective practice intervention had a greater impact in terms of empowering them to adopt an evidence-based practice mode!." | (Vachon, 2009) |
| MEASURING AND EVALUATING INTUITION | *Method* | "We present a new measure of intuition, the Types of Intuition Scale (TIntS), based on the theoretical view of intuition as three distinct types: holistic, inferential, and affective. " | (Pretz et al., 2014) |
|  |  | "The ethnographic and information-processing methods rely heavily on the reasoner’s awareness of how information is being used to make judgments. They are therefore limited in their ability to access the more unconscious, rapid and unrecoverable reasoning at the intuitive end of the continuum." | (Harries & Harries, 2001) |
|  |  | Gobet and Chassy expand on classical theories on chunking information in developing their Template Theory (TempT) where chunks and patterns of information are used in creating templates, or schema, from which information is readily encoded and accessed." | (Pickens et al., 2022) |
|  |  | "The reason that early research into clinical reasoning has not accurately accessed the full range of reasoning, from intuitive to analytic, appears to be due to methodological issues." | (Harries & Harries, 2001) |
|  | *Factors influencing measurement* | "Intuition is most accurate when experience has been acquired in a 'kind' environment." | (Pretz et al., 2014) |
|  |  | "In terms of clinical reasoning, then, therapeutic use of self appears important in understanding oneself, including one’s emotions, and may involve the use of intuition." | (Chaffey et al., 2012) |
|  |  | "The results also showed that legislation, pressure to conform, routine, tacit appraisal and intuition hindered the translation of research evidence into practice" . | (Vachon et al., 2010) |
|  |  | "Therapists needed to be aware of and understand their emotions to access intuition, to trust their emotions to act on them, and to use their emotions in problem solving and decision making" . | (Chaffey et al., 2012) |

**Table. 3.2 Thematic analysis of Intuition in clinical reasoning**

| Theme | *Sub-theme* | Examples | Authors |
| --- | --- | --- | --- |
| Intuition as Personalized Knowledge | *Experience* | "Intuition in decision making is most accurate when experience has been acquired in a 'kind' environment, one that provides clear and immediate feedback about the accuracy of judgments." | (Pretz et al., 2014) |
|  |  | "The participants in this study understood intuition to be knowledge without conscious reasoning. It is influenced by professional experience and the understanding and use of one’s own and others’ emotions." | (Chaffey et al., 2010) |
| . |  | "Their life experiences and personal interests enriched their evaluation of the app and demonstrated a depth beyond traditional 'early beginners.' Still, the ease in which the experienced practitioners engaged with the app, demonstrated depth of questioning, and anticipatory problem-solving illustrates the TempT Theory and use of intuition." | (Pickens et al., 2022) |
|  |  | "Most participants acknowledged the role of professional experience in supporting their intuition." | (Chaffey et al., 2010) |
|  |  | "Intuition plays a significant part in the decision-making process of skilled OT clinicians and has been attributed to their extensive experience and knowledge base" | (Rassafiani et al., 2009) |
|  |  | "Treating a sick person is an intrusive act of a professional and human nature. It is based both on acquired and proven knowledge as well as on accumulated clinical experience" | (Fried, 2020) |
|  |  | "Clinical knowledge based on discoveries attains validity in an empirical research process. Experience-based clinical knowledge, by its very nature, cannot be validated empirically and relies on intuition, analogies, common sense, and courage" | (Fried, 2020) |
|  |  | "Intuitive reasoning is often used in situations where information is incomplete or ambiguous. Experienced clinicians may use their intuition to quickly identify problems and potential solutions, but it is important to note that intuition should not be used as the sole basis for decision making." | (Harries & Harries, 2001) |
|  | *Expertise* | "Dreyfus and Dreyfus (1996) asserted that experts use intuition more than novices, with experts appearing to have an intuitive understanding of a situation and the appropriate actions they should take." | (Chaffey et al., 2012) |
|  |  | "Scientific rationality has proven inadequate as the sole approach to clinical reasoning. Grounding in theory does not assure expert practice, because while theory gives general principles, 'the uniqueness of each clinical situation requires judgment and improvisation." | Mekkes (2003) |
|  |  | "highly tacit and embodied knowledge, which is characteristic of the expert" | (Mattingly & Fleming, 1994) |
|  |  | "Reasoning through the complex home safety evaluation process, the expert practitioner engages multiple layers of knowledge with their practical knowledge, emotional intelligence, and intuition." | (Pickens et al., 2022) |
|  |  | "Artistry is inherent in this ability to individualize treatment by entering the client’s world, to contextualize the problem and to tailor intervention; it evolves from clinical experience and is essential in developing professional expertise" (Kielhofner, 1983)." | (Williams & Paterson, 2009) |
|  |  | "L'expertise permet ainsi le développement d'un mode de prise de décision intuitif qui est efficace et rapide pour intervenir dans les situations connues" | (Vachon, 2009) |
|  |  | "This expertise plays out in tailoring the knowledge message for the client’s perspective, as well as providing the client with a holistic picture of the range of applicable knowledge to enable informed choices." | (Metzler & Metz, 2010) |
|  |  | "Experienced OT clinicians seem to weigh different decision factors using intuition more effective" | (Rassafiani et al., 2009) |
|  |  | "Professional artistry is an integral and often unconscious component of professional expertise and is essential to good clinical decision-making for an individual client" (Higgs et al., 2001). " | (Williams & Paterson, 2009) |
|  |  | "much of our expertise becomes embodied in habitual ways of seeing and dealing with patients." | (Mattingly & Fleming, 1994) |
|  |  | Therapy represents a human encounter in which one person seeks help and the other, who possesses the necessary expertise and knowledge, provides it... To promote their patients' ability to function, therapists must have the courage to apply their evidence-based empirical knowledge and clinical experiential knowledge" | (Fried, 2020) |
|  |  | "Explorations of expert occupational therapists’ clinical reasoning also indicated that they practice intuitively" | (Chaffey et al., 2012) |
|  | *Occupational therapist's understanding of client and situation* | "Intuition helps therapists fill in the gaps and understand the experiences of the patient, especially in situations where quantitative comparison and measurement are not possible." | (Fried, 2020) |
|  |  | "Therapists frequently used intuitive reasoning rather than logical problem solving when individualizing treatment and trying to understand the client’s perspective." | (Leicht & Dickerson, 2002) |
|  |  | "Our aim is to establish a collaborative relationship with our patients, involving them in the decision-making process and empowering them to take an active role in managing their health." | (Hooper, 1997) |
|  |  | "Most participants spoke of knowing their client’s illness presentation and intuitively knowing when he or she was relapsing." | (Chaffey et al., 2010) |
|  |  | " She described this as having a “strong gut level” feeling in her interactions with clients, their families and even students and that she has an increasing confidence in the accuracy of these instincts. " | (Mekkes, 2003) |
|  | *Tacits Knowledge* | "In a recent grounded theory study of intuition among occupational therapists practicing in mental health, intuition was defined as knowledge that was immediate and accessed without a conscious awareness of reasoning. The study found that intuition was embedded in therapists’ clinical reasoning and was informed by tacit knowledge. " | (Chaffey et al., 2012) |
|  |  | "Clinical reasoning and tacit knowledge are crucial to the effective integration of research into the practice context." | (Metzler & Metz, 2010) |
|  |  | "Intuition is considered an important aspect of clinical expertise and is viewed as knowledge acquired through practice" | (Rassafiani et al., 2009) |
|  |  | "Instead, therapists rely on a unique and intuitive “knowing in action stance” to make clinical decisions (Mattingly, 1991). This process of knowing in action is based on individual’s normative judgments by which they recognize actions as right or wrong and, as a result, clinical reasoning becomes a tacit, subjective process. " | (Mekkes, 2003) |
|  |  | "Decision making at this stage was often based on tacit knowledge and intuition. " | (Vachon, 2009) |
| Intuition as a valuable decision-making tool | *rapid decision-making* | "The authors suggest that intuition is an important aspect of clinical reasoning and decision making in occupational therapy practice." | (Chaffey et al., 2012) |
|  |  | "The best decisions arising from the therapeutic process are based on the gamut of available knowledge with special regard for cross-validation, parsimony, and intuition." | (Metzler & Metz, 2010) |
|  |  | "This often difficult decision is influenced by many factors, such as clinical and social policies, resource constraints, intuition and professional expertise" | Grime (1990) |
|  |  | "Intuition plays a significant part in the decision-making process of skilled OT clinicians and has been attributed to their extensive experience and knowledge base" | (Rassafiani et al., 2009) |
|  |  | "Intuitive reasoning is often used in situations where information is incomplete or ambiguous. Experienced clinicians may use their intuition to quickly identify problems and potential solutions, but it is important to note that intuition should not be used as the sole basis for decision making." | (Harries & Harries, 2001) |
| Intuition as a source of creativity and joy | *Enriching experience through creativity* | "Intuition serves as a bridge when sensory and intellectual processes fail to inform adequately, enhancing creativity." | (Fried, 2020) |
|  |  | "Yes, creativity and change are risky. Anything can happen, including success. But inviting the intuitive into your hand therapy practice will bring you the experience of great joy. The process of therapy will become a vehicle for constant discovery of the knowledge and skills within you." | (Colditz, 2000) |
| . |  | "Here comes the intuition for help to fill in the gaps when we come to recognize the experience of the ‘other’ in the therapeutic space." | (Fried, 2020) |
|  |  | "Professional artistry is an integral and often unconscious component of professional expertise and is essential to good clinical decision-making for an individual client." | (Williams & Paterson, 2009) |
|  | *A source of joy* | "But inviting the intuitive into your hand therapy practice will bring you the experience of great joy. The process of therapy will become a vehicle for constant discovery of the knowledge and skills within you. You will have episodes of suddenly knowing something that you did not realize you knew." | (Colditz, 2000) |
| The limits of intuition | *Biases and errors* | The therapist in the above example described it as a ""trial-and-error"" process. It becomes evident, however, that the considerations involved in making this kind of small decision are quite elaborate. | (Pretz et al., 2014) |
|  |  | "Relying on gut instinct alone, often borne out of laziness or arrogance or both, will produce bad decisions." | (Higgs, 2019) |
|  |  | "Bias and intuition, e.g. knowledge of client or referrer, given diagnosis, accumulated knowledge, local knowledge, e.g. from clinical and professional knowledge, local contacts, training and expertise." | Grime (1990) |
|  |  | "Although intuition is an important aspect of clinical reasoning and decision-making, it is not infallible. Intuition can be influenced by biases, emotions, and other factors that may lead to errors in judgement | (Higgs, 2019) |
|  |  | "The result is a vague perception of coherence which is not explicitly describable but instead embodied in a gut feeling or an initial guess, which subsequently biases thought and inquiry." | (Higgs, 2019) |
|  | *Limitations in specific contexts* | "Intuition is particularly important in situations where the facts are unclear, the problem lacks clarity, or the issues cannot be decomposed." | (Higgs, 2019) |
|  |  | "Change and the expression of one's creativity as a therapist, especially in this litigious American society, can be dangerous in the clinical environment...I would like to salute two hand therapists who have given themselves permission to listen to their intuition." | (Colditz, 2000) |
|  |  | "Most occupational therapists who argue for occupational therapy professional management feel that clinical judgement, based on the occupational therapist's professional expertise, is required to interpret the additional cues present in each referral in order to identify the potential needs of the individual, for example, diagnosis, prognosis, family and geographical location." | Grime (1990) |
|  |  | "Le défi de ces participantes intuitives était d'apprendre à prendre un temps d'arrêt pour réfléchir et planifier leurs actions, ce que le cadre du groupe réflexif leur a offert." | (Vachon, 2009) |
